# Supplementary material for: Tartrazine clears live cells while preserving viability at high refractive indices and osmolality
Source: bioRxiv. 2026 Apr 13:2026.04.09.717314. Preprint. [Version 1] doi: 10.64898/2026.04.09.717314 (PMC13104990; doi:10.64898/2026.04.09.717314)
Supplement: Supplement 3 [file media-3.pdf]

Supplementary Materials for

Tartrazine clears live cells while preserving viability at high refractive indices and osmolality

Xuandi Hou,[1,2] Sa Cai,[1,2] Han Cui,[1,2] Zhongyu Liu,[1,2] Su Zhao,[1,2] Ling-Yi Zhang,[1,2] Ani Baghdasaryan,[1,2] Victoria Crunkleton,[1,2] Mark L. Brongersma[1,3,4*] and Guosong Hong[1,2*]

[1]Department of Materials Science and Engineering, Stanford University, Stanford, CA, USA

[2]Wu Tsai Neurosciences Institute, Stanford University, Stanford, CA, USA

[3]Geballe Laboratory for Advanced Materials, Stanford University, Stanford, CA, USA

[4]Department of Applied Physics, Stanford University, Stanford, CA, USA

*To whom correspondence may be addressed. Email: markb29@stanford.edu, guosongh@stanford.edu

Table of Contents

Figures S1–5……………………………………………………………………………….2

Tables S1……………………………………………………………………………………..7

Movie captions S1–2…………………………………………………………………………8

Supplementary figures

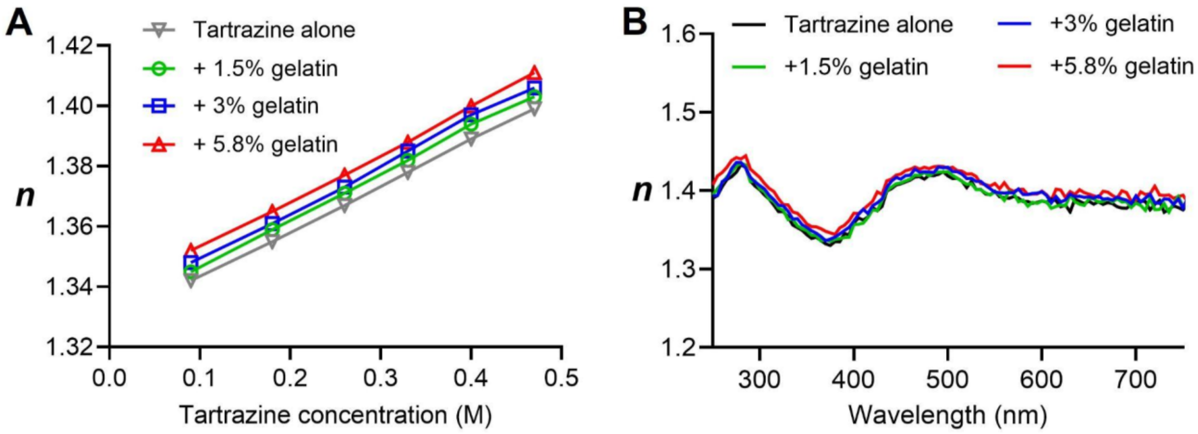

Figure S1. RI dependence on tartrazine and gelatin concentrations. (**A**) Dependence of the real refractive index (*n*) measured by an Abbe refractometer on the concentration of tartrazine with different gelatin concentrations. (**B**) *n* of 0.47 M tartrazine solutions containing different concentrations of gelatin measured as a function of wavelength by ellipsometry.

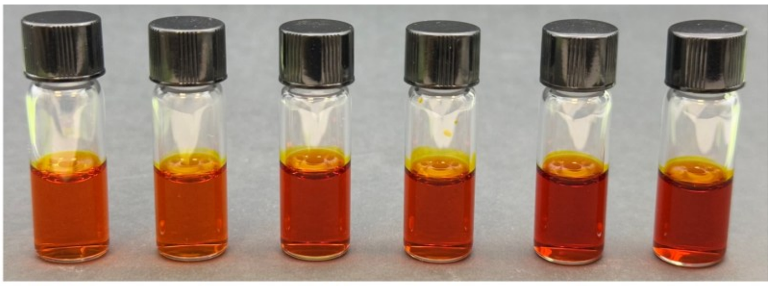

Tartrazine (M)	0.18	0.18	0.33	0.33	0.47	0.47
Gelatin (% w/w)	0	5.8	0	5.8	0	5.8

Figure S2. Photographs of solutions containing varying concentrations of tartrazine and gelatin.

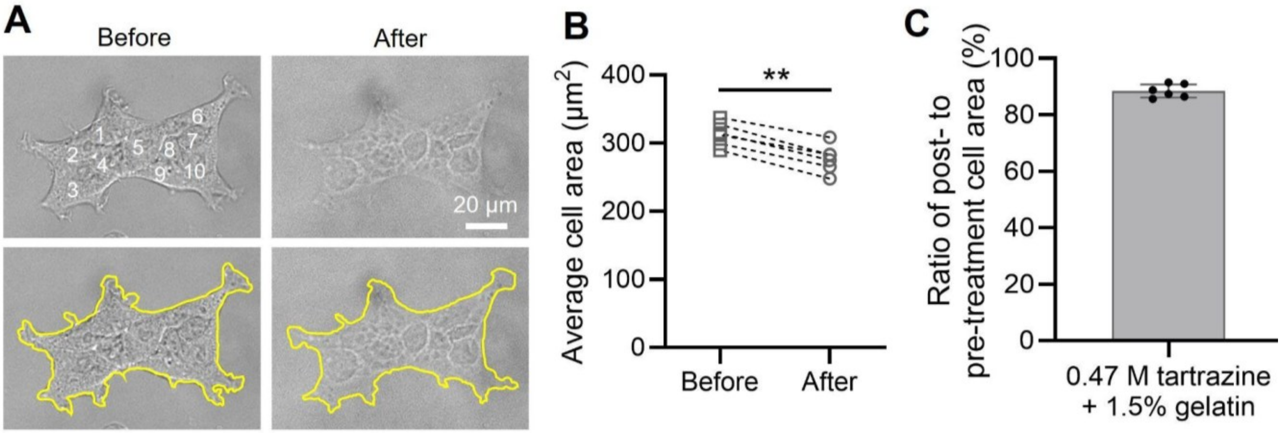

Figure S3. Cell morphology and size changes in response to the tartrazine solution containing 1.5% gelatin. (**A**) HEK cells immersed in a solution containing 0.47 M tartrazine and 1.5% w/w gelatin. (**B**) Quantification of the average cell area following tartrazine/gelatin treatment in **A**. (**C**) Quantification of the percent cell area change following tartrazine/gelatin treatment in **A**. **$p < 0.01$, as determined by unpaired two-tailed t-tests. $n = 6$ biological replicates for panels **B** and **C**. Data in panels **C** is presented as mean ± SD.

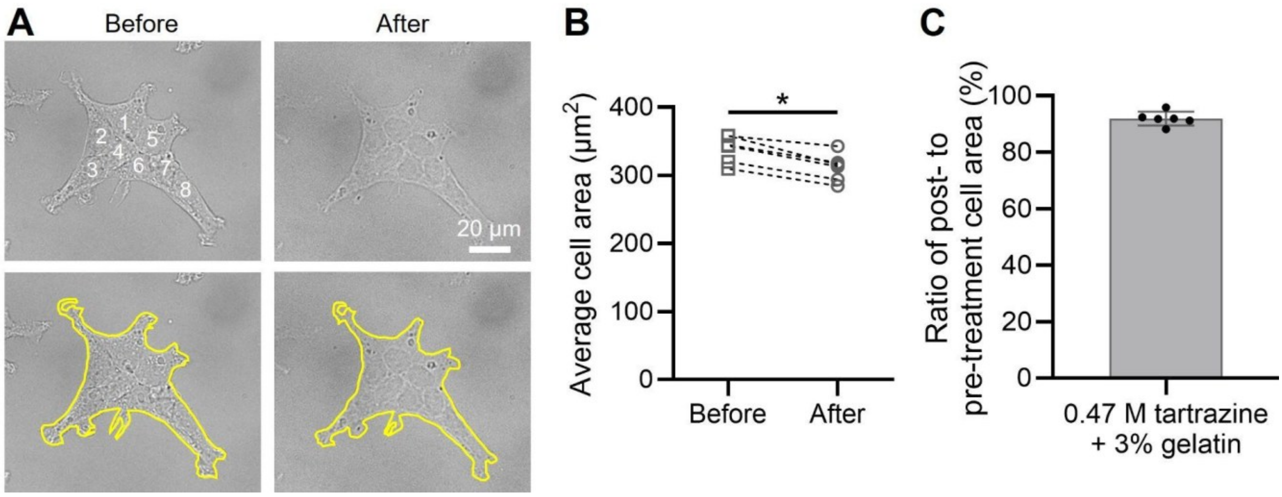

Figure S4. Cell morphology and size changes in response to the tartrazine solution containing 3% gelatin. (**A**) HEK cells immersed in a solution containing 0.47 M tartrazine and 3% w/w gelatin. (**B**) Quantification of the average cell area following tartrazine/gelatin treatment in **A**. (**C**) Quantification of the percent cell area change following tartrazine/gelatin treatment in **A**. *$p < 0.05$, as determined by unpaired two-tailed t-tests. $n = 6$ biological replicates for panels **B** and **C**. Data in panels **C** is presented as mean ± SD.

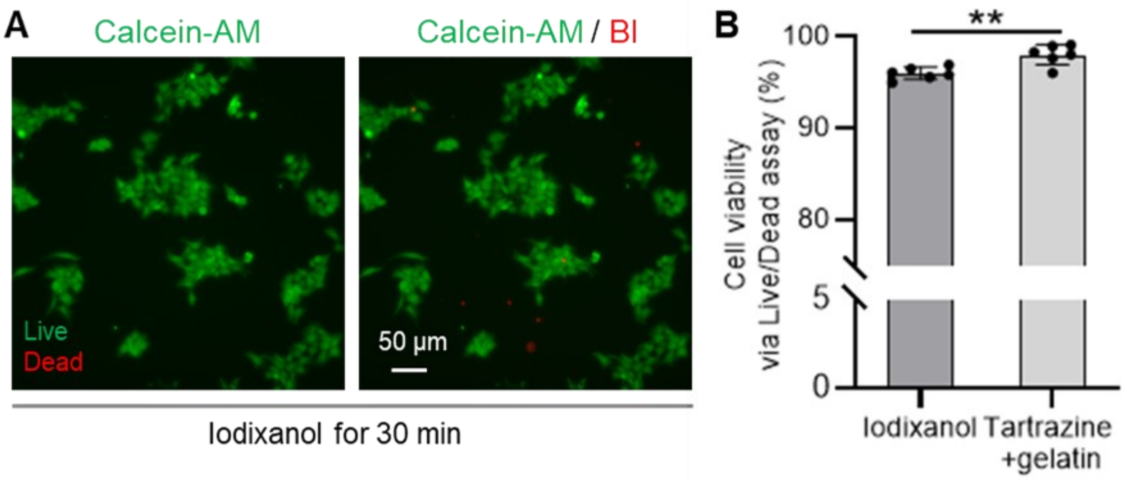

Figure S5. In vitro cell viability assessment of another clearing agent, iodixanol. (**A**) Representative images of live/dead staining in HEK cells after 30 minutes of incubation with iodixanol (RI 1.41). Live cells are shown in green, and dead cells in red. (**B**) Quantification of cell viability following incubation, based on live/dead staining. Data from the 30-minute incubation with 0.47 M tartrazine and 5.8% gelatin is also presented in **Fig. 4B**. **$p < 0.01$, as determined by unpaired two-tailed t-tests. Data are presented as mean ± SD in panel **B** for $n = 6$ biological replicates.

Table S1. Chemical compositions of different tartrazine/gelatin solutions.

	Tartrazine (mg)	Water (mg)	Gelatin (mg)
0.18 M tartrazine + 5.8% gelatin	100	1000	67.7
0.33 M tartrazine + 5.8% gelatin	200	1000	73.8
0.47 M tartrazine + 5.8% gelatin	300	1000	80
0.47 M tartrazine	300	1000	0
0.47 M tartrazine + 1.5% gelatin	300	1000	19.8
0.47 M tartrazine + 3% gelatin	300	1000	40.2

Movie captions

Movie S1. HEK cells lose membrane contrast upon addition of 0.47 M tartrazine and 5.8% gelatin, with no significant cell shrinkage observed. The video is shown at 5× real-time speed.

Movie S2. HEK cells lose membrane contrast upon addition of 0.47 M tartrazine, resulting in noticeable cell shrinkage. The video is displayed at 5× real-time speed.
